# The Clinical Promise of Microalgae in Rheumatoid Arthritis: From Natural Compounds to Recombinant Therapeutics

**DOI:** 10.3390/md21120630

**Published:** 2023-12-07

**Authors:** Edoardo Andrea Cutolo, Roberto Caferri, Rosanna Campitiello, Maurizio Cutolo

**Affiliations:** 1 Laboratory of Photosynthesis and Bioenergy, Department of Biotechnology, University of Verona, Strada le Grazie 15, 37134 Verona, Italy; roberto.caferri@univr.it; 2 Research Laboratory and Academic Division of Clinical Rheumatology, Department of Internal Medicine, IRCCS San Martino Polyclinic Hospital, University of Genoa, Viale Benedetto XV, 6, 16132 Genoa, Italy; rosannacampitiello@hotmail.it (R.C.);

**Keywords:** photosynthesis, polyunsaturated fatty acids, carotenoids, oxylipins, xanthophylls, antioxidants, functional foods, synthetic biology, genetic engineering, inflammation, rheumatoid arthritis, autoimmunity, docosahexaenoic acid (DHA), eicosapenteanoic acid (EPA), astaxanthin, rheumatology, interleukins, chloroplast, molecular pharming, novel foods, bioeconomy

## Abstract

Rheumatoid arthritis (RA) is an invalidating chronic autoimmune disorder characterized by joint inflammation and progressive bone damage. Dietary intervention is an important component in the treatment of RA to mitigate oxidative stress, a major pathogenic driver of the disease. Alongside traditional sources of antioxidants, microalgae—a diverse group of photosynthetic prokaryotes and eukaryotes—are emerging as anti-inflammatory and immunomodulatory food supplements. Several species accumulate therapeutic metabolites—mainly lipids and pigments—which interfere in the pro-inflammatory pathways involved in RA and other chronic inflammatory conditions. The advancement of the clinical uses of microalgae requires the continuous exploration of phytoplankton biodiversity and chemodiversity, followed by the domestication of wild strains into reliable producers of said metabolites. In addition, the tractability of microalgal genomes offers unprecedented possibilities to establish photosynthetic microbes as light-driven biofactories of heterologous immunotherapeutics. Here, we review the evidence-based anti-inflammatory mechanisms of microalgal metabolites and provide a detailed coverage of the genetic engineering strategies to enhance the yields of endogenous compounds and to develop innovative bioproducts.

## 1. Introduction

Chronic inflammation is a defining feature of autoimmune diseases, a group of conditions in which immunological self-tolerance is disturbed due to the recognition of autoantigens by immune cells. Rheumatoid arthritis (RA), the most common chronic inflammatory arthropathy [1,2], is a systemic autoimmune disorder affecting the synovial joints, with a higher incidence in women [3]. RA displays a complex pathophysiology involving the upregulation of pro-inflammatory mediators (interleukins, ILs) and enhanced production of reactive oxygen species (ROS) [4,5]. Both genetic and modifiable lifestyle factors contribute to the risk of RA predisposition [6,7], with diet highly influencing disease activity [8,9]. In particular, a high antioxidant intake is known to reduce onset risk and to ameliorate the clinical course of the disease [10], therefore, the identification of new sources of antioxidant and anti-inflammatory molecules is of high clinical relevance.

Microalgae are photosynthetic prokaryotes and eukaryotes adapted to diverse environments, including extreme habitats [11,12], which are consumed in human nutrition as sources of proteins and other bioactive compounds [13,14,15,16,17]. Several species are non-toxic producers of essential vitamins, lipids, and pigments of therapeutic value [18,19,20,21,22], which could be employed as complementary agents in the management of chronic inflammatory diseases. Moreover, the fast life cycle and light-powered autotrophic metabolism of microalgae allows for large-scale cultivation with lower inputs compared with heterotrophic microorganisms [23].

In this review, we summarize the evidence-based putative interference of microalgal compounds in pro-inflammatory pathways involved in the pathogenesis of RA and discuss how bioprospecting for novel pharmacologically relevant strains, and their domestication, can advance the clinical use of photosynthetic microorganisms. Lastly, we provide an update on the available genetic engineering strategies to enhance the production of endogenous microalgal metabolites and introduce emerging approaches to achieve light-driven conversion of CO_2_ into high-value recombinant biopharmaceuticals.

### Pathogenesis and Mediators of Rheumatoid Arthritis

**Figure 1:**
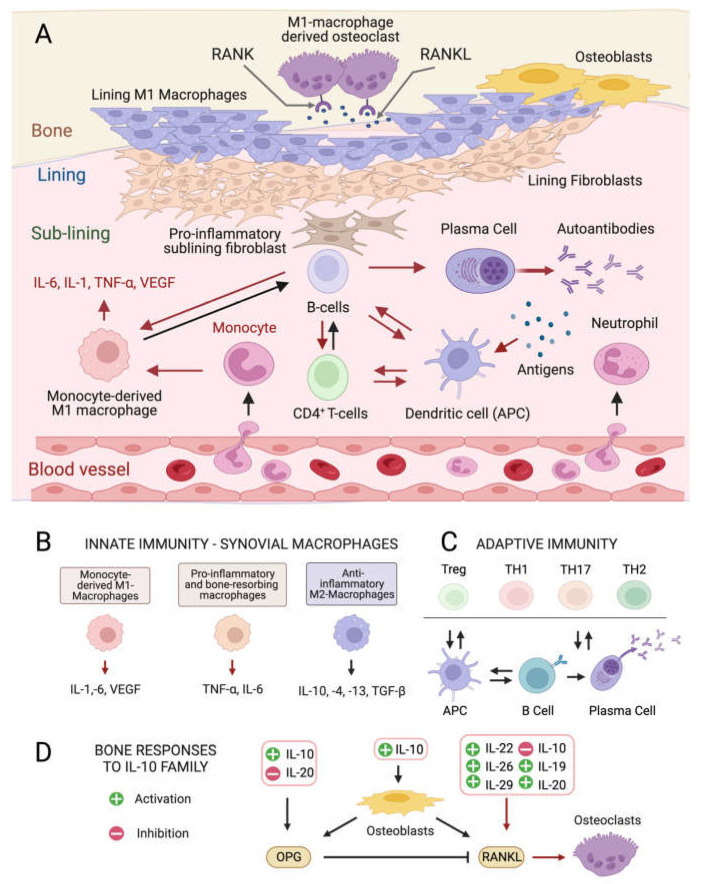
Cellular composition of the synovial membrane, its interaction with bone tissue and immune cell types, and related mediators involved in inflamed RA joints. (**A**) In healthy conditions, the synovium consist of a thin lining layer of lining fibroblasts in association with lining M1 directly exposed to the bone tissue. The underlying sub-lining layer is a connective tissue enriched in blood vessels, adipocytes, fibroblasts, and both innate and adaptive immune cells. The inflamed RA synovium is characterized by a hyperplastic lining layer surrounded by proinflammatory sub-lining fibroblasts and a massive infiltration of B-cells, monocyte-derived macrophages, autoantibody-secreting plasma cells, and differentiated cytotoxic effector memory CD4^+^ T-cells in the sub-lining layer. The secretion of pro-inflammatory interleukins (IL-1: interleukin 1; IL-6: interleukin 6; TNF- α: tumor necrosis factor-alpha) by activated immune cells stimulates the production of the soluble cytokine Mediator Receptor Activator of Nuclear Factor Kappa-Β Ligand (RANKL), which binds to its receptor RANK on monocytes and macrophages causing their differentiation into bone-resorbing osteoclasts. Red arrows indicate pro-inflammatory processes. (**B**) Interleukin (IL) isoforms produced by different types of synovial innate immune cells and macrophages (IL-6: interleukin 6; IL-1: interleukin 1; TNF-α: tumor necrosis factor-alpha; TNF-β: tumor necrosis factor-beta; VEGF: vascular endothelial growth factor). (**C**) Differentiation and interconversion of adaptive immune cells (Treg: regulatory T cells; TH1: T helper 1 cells; TH 17: T helper 17 cells; TH2: T helper 2 cells; B cells; APCs: antigen-presenting cells). (**D**) Effects of secreted ILs on bone-remodeling processes depending on the osteoprotegerin (OPG)–RANKL axis, which regulates the differentiation of osteoclasts in bone-resorbing osteoclasts. Figures created with BioRender.com, accessed on 15 November 2023.

Although the exact etiology of RA remains unknown, the balance between immune cells and the production of inflammatory ILs in the connective tissue that lines the joint capsule (synovium) is altered in the disease onset and progression [4,5]. The healthy synovium consists of a thin lining layer of fibroblasts covering a connective tissue surrounded by blood vessels and enriched in fibroblasts, and innate and adaptive immune cells: the sub-lining layer [24] (Figure 1A). In RA, the lining layer is hyperplastic while the sub-lining layer is infiltrated with B-cells, monocyte-derived macrophages, autoantibody-secreting plasma cells, and differentiated cytotoxic CD4^+^ T-cells involved in the breakdown of tissue tolerance [25,26,27]. The release of pro-inflammatory ILs by monocyte-derived M1 macrophages, and osteoclast activation cause progressive bone resorption [28], and autoantibodies produced by differentiated plasma cells further contribute to joint damage [29].

At the intracellular level, three main pro-inflammatory signaling kinase cascades responding to soluble mediators are involved in RA, all being influenced by microalgal metabolites: the Nuclear Factor Kappa-Β (NF-kB)-mediated pathway [30], the Janus kinase 2/Signal Transducers and Activators of Transcription 3 (JAK2/STAT3) pathway [31], and the Jun N-terminal kinase (JNK)/p38 Mitogen-Activated Protein Kinase (p38 MAPK) pathway [32], the latter being predominant in the lining layer and endothelial cells.

Pro-inflammatory ILs (Figure 1D) [33] stimulate the production of the soluble cytokine Mediator Receptor Activator of Nuclear Factor Kappa-Β Ligand (RANKL) by immune cells. RANKL binds its receptor RANK on monocytes and macrophages causing their differentiation into bone-resorbing osteoclasts [34]. Other ILs produced by anti-inflammatory M2 macrophages positively influence the osteoprotegerin (OPG)–RANKL ratio, promoting bone homeostasis [35] (Figure 1D), while the IL-10 family (Figure 1D) appears to play dual roles. Some members stimulate osteogenesis and suppress the synthesis of pro-inflammatory mediators like IL-6, Tumor Necrosis Factor-α (TNF-α), and Vascular endothelial growth factor (VEGF) [36], while others activate the NF-kB and MAPK pathways, stimulating TNF-α, IL-1β, and RANKL production by synovial fibroblasts, promoting osteoclastogenesis.

In conclusion, given the diversified nature of factors regulating the balance between pro- and anti-inflammatory mediators in RA, there is a strong interest in discovering novel molecules to be used as complementary tools alongside conventional therapies.

## 2. Anti-Inflammatory and Immunomodulatory Metabolites from Microalgae

### 2.1. Carotenes and Xanthophylls

**Figure 2:**
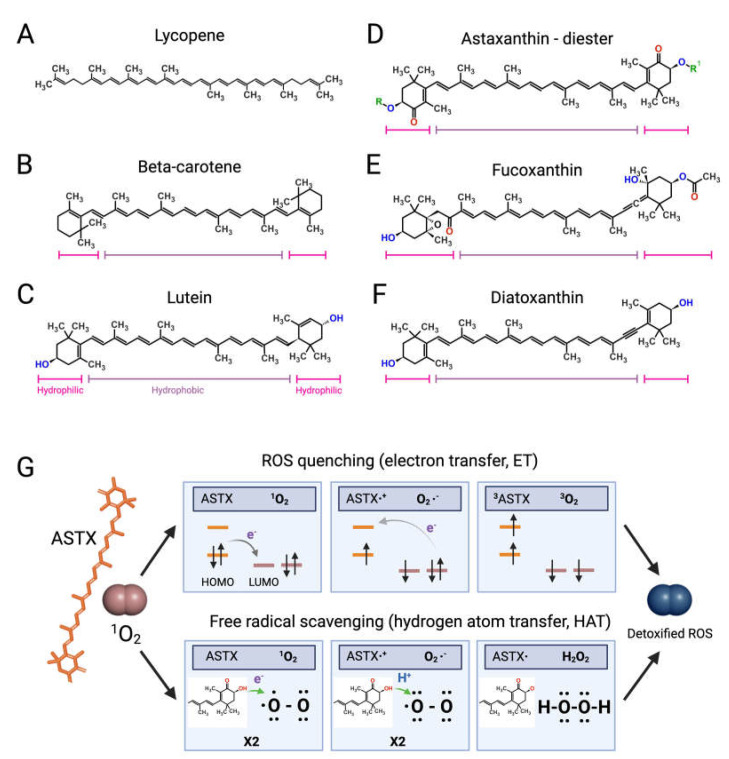
Structures of microalgal carotenoids and ROS detoxification mechanisms of astaxanthin. Lycopene (**A**), beta-carotene (**B**), lutein (**C**), astaxanthin (ASTX, (**D**)), fucoxanthin (**E**), and diatoxanthin (**F**). Pink and purple bars indicate the hydrophobic and hydrophilic regions of the molecules, respectively; in red, the oxygen of the keto groups, and in blue, the oxygen of the carboxylic groups; in green, the R and R’ functional groups of astaxanthin. Panel (**G**) outlines the two main routes of ASTX-mediated singlet molecule oxygen (^1^O_2_) detoxification. The top pathway is based on an electron transfer process involving: (i) the formation of a weakly bound ASTX-^1^O_2_ complex followed by direct electron transfer from the highest occupied molecular orbital (HOMO) of ASTX to the lowest unoccupied molecular orbital (LUMO) of singlet oxygen (^1^O_2_), and the formation of radicals; (ii) a reverse reaction restoring the electron distribution between the two molecules. The overall process converts ^1^O_2_ to its triplet unreactive form (^3^O_2_) upon spin inversion, while ASTX is restored from ^3^ASTX via internal conversion [55]. The bottom pathway shows the free radical scavenging activity based on a two-step transfer involving both an electron and proton (H^+^) from ASTX to ^1^O_2_. The formed hydrogen peroxide is readily removed by peroxidase enzymes while ASTX is spontaneously restored by ascorbate. These mechanisms of action are iterative, meaning that a single ASTX molecule can perform multiple ROS detoxification cycles. Figures created with BioRender.com, accessed on 15 November 2023.

Like bacteria, fungi, and plants, microalgae synthetize C40 lipophilic pigments consisting of a polyene chain of conjugated double bonds (Figure 2) with terminally linked ionone rings known as carotenoids [37,38]. β-carotene (Figure 2B)—a structural element of photosystems [39]—and the antioxidant lycopene (Figure 2A) are anti-inflammatory carotenes [40,41] usually introduced in the diet with carrots (*Daucus carota*) and tomatoes (*Solanum lycopersicum*), respectively, although present also in microalgae [42].

Xanthophylls are oxygenated carotenoids containing hydroxyl and ketone groups in the ionone rings, which serve different functions in phototrophs. The non-ketolated xanthophyll lutein (Figure 2C) participates in light-harvesting and photoprotection, while the ketocarotenoid astaxanthin (ASTX, Figure 2D) scavenges harmful ROS generated by photosynthetic electron transport under excess light [43]. Lutein is an anti-inflammatory carotenoid [44] abundantly found in green leafy vegetables and egg yolk, while ASTX is a potent antioxidant uniquely synthetized by a few microalgal species.

Abiotic stresses induce a hypercarotenogenic response in several chlorophytes, including the halophile *Dunaliella salina* (Chlorophyceae), which overaccumulates β-carotene in lipid bodies (plastoglobules) inside the chloroplast [45], and in the freshwater species *Haematococcus pluvialis* (Haematococcaceae), which forms haematocysts filled with ASTX-rich cytoplasmic lipid droplets [46]. Other microalgal xanthophylls with anti-inflammatory and immunomodulatory properties are fucoxanthin and diatoxanthin produced by several diatoms (stramenopiles) and by the haptophyte *Tisochrysis lutea* (Coccolithophyceae) (Figure 2E,F) [47,48]. As discussed in the following paragraphs, carotenoids appear to interfere in all major pro-inflammatory pathways implicated in the onset and progression of RA.

#### Astaxanthin: The Red Gold of Algae

With recognized safety for human consumption [49], approved Novel Food status [50], and an established role in promoting bone homeostasis in degenerative skeletal diseases [51], ASTX is the microalgal pigment of highest biopharmaceutical value.

Clinical studies have shown that ASTX intake reduces the levels of systemic inflammatory biomarkers [52,53] and potentiates the pain-relieving effect of anti-inflammatory therapies [54]. The pharmacological effects of ASTX derive from its strong antioxidant-activity mediated via ROS quenching [55] (Figure 2G, top panel) and direct free radical scavenging [56,57] (Figure 2G, bottom panel). This amphipathic molecule is symmetrically arranged within the lipid bilayer [58], thus exerting antioxidant activity on both intra- and extracellular environments. Notably, the higher number of hydroxyl groups compared with other carotenoids confers to ASTX superior ROS-detoxifying capacity [59]. Early studies showed that ASTX suppressed ROS production [60,61,62,63] and secretion of pro-inflammatory ILs by cultured human-activated monocytes [64]. Moreover ASTX stimulated the expression of ROS-scavenging enzymes in chondrocytes challenged with IL-1β [65], and inhibited pro-inflammatory and osteoclastogenic gene expression in macrophages challenged with RANKL [66]. Lastly, the administration of ASTX promoted cartilage health in animal models of arthritis and osteoasthritis [67,68,69].

Notably, the esterified biological form of ASTX displays higher bioavailability compared with synthetic ester-free derivatives [70,71,72,73], suggesting the need to improve its production from natural sources. However, a major limitation to the clinical use of ASTX lies in its low solubility in gastrointestinal fluids [74], requiring encapsulation in polysaccharide, lipid, and protein nanoparticles to enhance its delivery and release [75,76,77,78,79,80].

### 2.2. Anti-Inflammatory Mechanisms of Action of Astaxanthin and Other Carotenoids

#### 2.2.1. NF-κB Pathway

ASTX and β-carotene interfere with the NF-κB pathway blocking the translocation of the NF-κB transcription factor to the nucleus, thereby suppressing ROS and pro-inflammatory gene expression. This effect is likely mediated through targeting the Inhibitor of the NF-κB γ subunit (IKK-γ) of the IkB kinase complex [81,82,83,84]. This prevents the phosphorylation and subsequent proteasome-mediated degradation of the IkBα binding factor, which abolishes the release of NF-κB [30]. A similar inhibitory effect has been proposed for fucoxanthin and diatoxanthin [85,86].

The Mitogen- and Stress-activated protein Kinase-1 (MSK1) is a nucleus-localized factor, which activates the NF-κB pathway [87] and the transcriptional regulator cAMP-responsive Element-Binding Protein (CREB) [88]. Phosphorylated CREB binds CREB-Responsive Elements (CRE) promoting pro-inflammatory gene expression [89]. These events are suppressed by ASTX, which inhibits MSK1 autophosphorylation [90]. Lastly, in silico simulations suggested that ASTX and β-carotene extracellularly interact with IL-6 and TNF-α, preventing their binding to membrane receptors [91]. ASTX may also interact with the NF-κB-Inducing Kinase (NIK) and block the phosphorylation of the IKK-α subunit of the IkB α kinase complex, suppressing the NF-κB pathway [92].

#### 2.2.2. JAK2/STAT3 and JNK/p38 MAPK Pathways

β-carotene and ASTX further modulate the pro-inflammatory pathways mediated by the JNK/p38 MAPK [93] and JAK2/STAT3 kinases [84,94], the latter responding to IL-6 in the pathogenesis of RA and osteoarthritis [31,95]. Phosphorylation of STAT3 dimers by JAK2 induces nuclear translocation and the differentiation of CD4^+^ T cells into the highly reactive T helper 17 (*Th17*) cell type [96]. TNFs and IL-1 activate the JNK/p38 MAPK pathway starting a phosphorylation cascade ending with JNK/p38 MAPK nuclear translocation [97], and phosphorylation of pro-inflammatory transcription factors (ELK1, MEF2, ATF2, and STAT1) [98] and of the MAPK-activated kinase 2 (MK2), which, in turn, targets the tristetrapolin (TTP) factor, promoting stabilization of IL mRNAs [99]. These oxidant-sensitive inflammatory pathways are also modulated by lutein [100], as reported using extracts enriched in this xanthophyll from different species of the chlorophyte genus *Tetraselmis* (Chlorodendrophyceae) [101].

#### 2.2.3. Other Pro-Inflammatory Pathways Targeted by Microalgal Carotenoids

Mitochondrial disfunction is a key pathogenic driver in RA [102], and ASTX was reported to attenuate organellar ROS production in human chondrocytes treated with IL-1β [69]. In addition to suppressing pro-inflammatory pathways, ASTX is also suggested to promote cartilage homeostasis via the transcriptional regulator nuclear factor-erythroid 2-related factor 2 (Nrf2) [68,93]. ASTX is suggested to stabilize and promote the nuclear translocation of Nfr2, which binds so-called antioxidant response elements (AREs), enhancing the expression of anti-inflammatory and ROS-detoxifying genes [103,104].

### 2.3. Lipids and Their Derivatives

A hallmark of RA is the altered fatty acid profile of the synovium [105,106], while the intake of polyunsaturated fatty acids (PUFAs) correlates with joint health and mitigates the risk of RA onset [107]. Microalgal mass cultivation is a more sustainable way to derive functional lipids compared with cold water fish [108,109,110,111,112]. Phytoplankton occupies the lowest trophic level in oceans and freshwater basins, representing the primary PUFAs producer in aquatic food webs [113]. Global warning and ocean acidification are predicted to affect phytoplankton ecology [114,115], thus reducing PUFAs availability to higher trophic levels and, eventually, putting at risk the supply for human nutrition [116]. Moreover, upon stress acclimation, microalgae synthetise a wider range of anti-inflammatory and immunomodulatory lipids compared to animals [117,118,119,120,121].

#### 2.3.1. Long-Chain Polyunsaturated Fatty Acids

**Figure 3:**
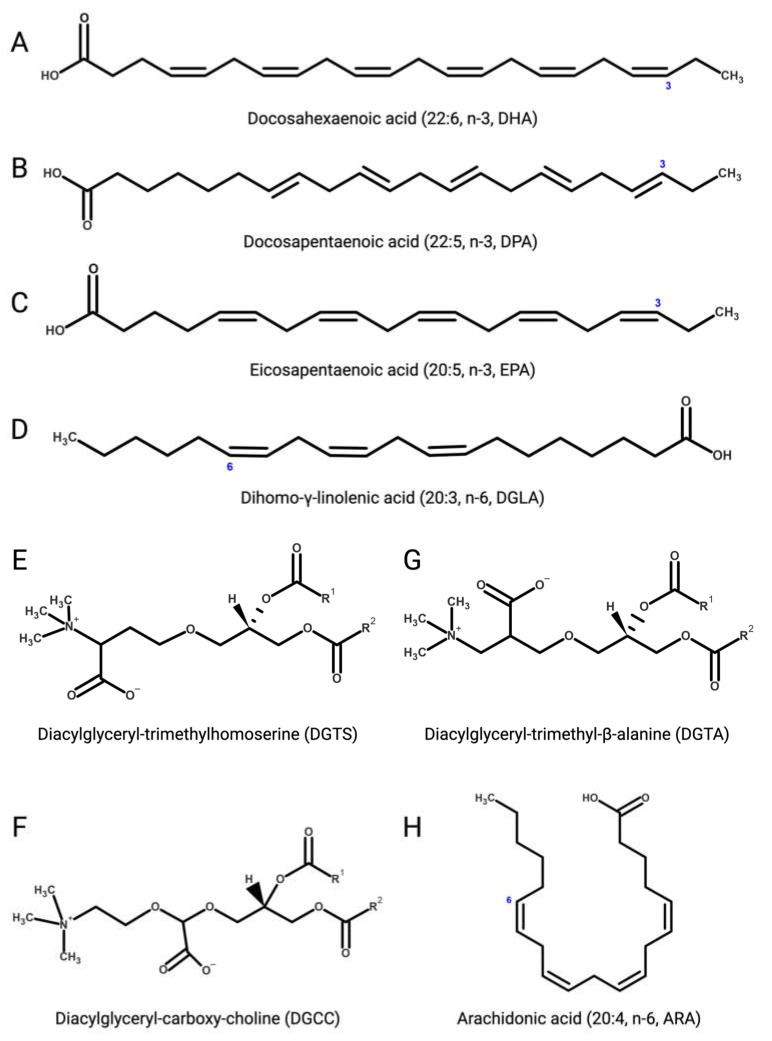
(**A**) Docosahexaenoic (DHA, 22:6, n-3); **(B)** docosapentaenoic (DPA, 22:5, n-3); (**C**) eicosapentaenoic (EPA, 20:5, n-3); (**D**) dihomo-γ-linolenic (DGLA, 20:3, n-6); (**E**) 1,2-diacylglyceryl-3-*O*-4’-(*N,N,N*-trimethyl)-homoserine (DGTS); (**F**) 1,2-diacylglyceryl-3-*O*-carboxy-(hydroxymethyl)-choline (DGCC); (**G**) 1,2-diacylglyceryl-3-*O*-2’-(hydroxymethyl)-(*N,N,N*-trimethyl)-β-alanine (DGTA); (**H**) arachidonic (ARA, 20:4, n-6). Figures created with BioRender.com, accessed on 15 November 2023.

Several microalgae accumulate very long-chain PUFAs [122,123], including the omega-3 (ω-3, n-3) PUFAs [124] α-linolenic (18:3), docosahexaenoic (DHA, 22:6, n-3, Figure 3A)**,** docosapentaenoic (DPA, n-3, 22:5, Figure 3B), and eicosapentaenoic (EPA, 20:5, n-3, Figure 3C) acids but also ω-6 PUFAs like arachidonic (ARA, 20:4, n-6, Figure 3H), γ-linolenic (18:3), linoleic (18:2), and dihomo-γ-linolenic (DGLA, 20:3, n-6, Figure 3D) acids. These molecules are biosynthetic precursors of anti-inflammatory signaling molecules and interfere with pro-inflammatory pathways [125,126].

The administration of lipids from the DGLA-hyperproducing freshwater chlorophyte *Lobosphaera incisa* (Trebouxiophyceae) suppressed the expression of the NF-κB pathway-related genes in an animal model of chronic inflammation [127]. Similarly, lipid extracts from the haptophyte *Pavlova lutheri* (Prymnesiophyceae), an EPA- and DHA-hyperaccumulating strain, inhibited IL-6 and TNF-α production in activated human macrophages, possibly through suppressing the NF-κB pathway [128].

Several heterotrophic marine microorganisms are strong DHA producers, such as the dinoflagellate *Crypthecodinium cohnii* (Dinophyceae) and, above all, the thraustochytrids protists *Thraustochytrium* spp., *Aurantiochytrium* (formerly *Schizochytrium) limacinum* (Labyrinthulomycetes) [129,130,131,132,133], and related genera [134]. Although these microorganisms cannot exploit light energy to drive their metabolism, they are capable of fermenting plant biomass hydrolysates, affording cost-effective heterotrophic cultivation using renewable feedstocks [135,136,137,138,139,140,141]. Arguably, the most valuable heterotrophic sources of DHA are *Schizochytrium* spp., which recently obtained Novel Food status [142,143]. Notably, a recent human clinical trial investigated the supplementation of an EPA- and DHA-enriched oil from a *Schizochytrium* sp. in RA patients, reporting beneficial effects on joint health and the blood levels of inflammatory markers [144].

#### 2.3.2. Betaine Lipids

Betaine lipids are anti-inflammatory and immunomodulatory glycerolipids in which the phosphate and carbohydrate moieties attached to the glycerol backbone are replaced with positively charged ether-bond betaine groups. Betaine lipids are widely distributed in all clades of eukaryotic microalgae, where they act as acyl group donors upon membrane lipid remodelling and during the accumulation of storage neutral lipids [145,146].

Betaine lipids derive from the turnover of membrane phospholipids under abiotic stresses, mainly temperature and nutrient starvation [147,148,149]. The betaine lipid 1,2-diacylglyceryl-3-*O*-4’-(*N,N,N*-trimethyl)-homoserine (DGTS, Figure 3E) is the most abundant betaine lipid in microalgae, followed by 1,2-diacylglyceryl-3-*O*-carboxy-(hydroxymethyl)-choline (DGCC, Figure 3F), and 1,2-diacylglyceryl-3-*O*-2’-(hydroxymethyl)-(*N,N,N*-trimethyl)-β-alanine (DGTA, Figure 3G), with evidence of DGTS-mediated inhibition of the IKK-β kinase causing suppression of the NF-κB pathway and secretion of pro-inflammatory ILs by Th1 and Th2 cells, as recently reported with extracts from the oleaginous chlorophyte *Chromochloris zofingiensis* (Chlorophyceae) [150].

#### 2.3.3. Oxylipins

The eicosanoids, prostaglandins and leukotrienes, and the thromboxanes, are hormone-like oxygenated metabolites of C20 fatty acids involved in the modulation and resolution of inflammation in the RA synovium [106]. In mammals, oxylipins are produced by the enzyme phospholipase A2 via release of *sn-2* PUFAs from membrane phospholipids. Typical oxylipin precursors are linoleic, α-linolenic, and ARA, which are substrates of cyclooxygenases, lipoxygenases, and cytochrome P450 enzymes, respectively [151].

Several microalgal species are known to accumulate prostaglandin-like oxylipins [152,153,154]. Their synthesis can occur either enzymatically via animal-like biosynthetic pathways [155], as in diatoms *Skeletonema marinoi* and *Thalassiosira rotula* (Bacillariophyceae) [156,157,158], or via spontaneous oxidation of ARA, EPA, and DHA in *T. lutea* [159]. The latter, known as isoprostanoids, are functional lipids which regulate bone health through preventing osteoclast differentiation [160].

The oxylipins isolated from the chlorophytes *Chlamydomonas debaryana* (Chlorophyceae) and *Nannochloropsis gaditana* inhibited TNF-α production in cultured macrophages [161], while the oral administration of biomass of the former suppressed the production of pro-inflammatory ILs in an animal model of chronic inflammation, possibly through inhibiting the NF-κB pathway [162,163]

**Figure 4:**
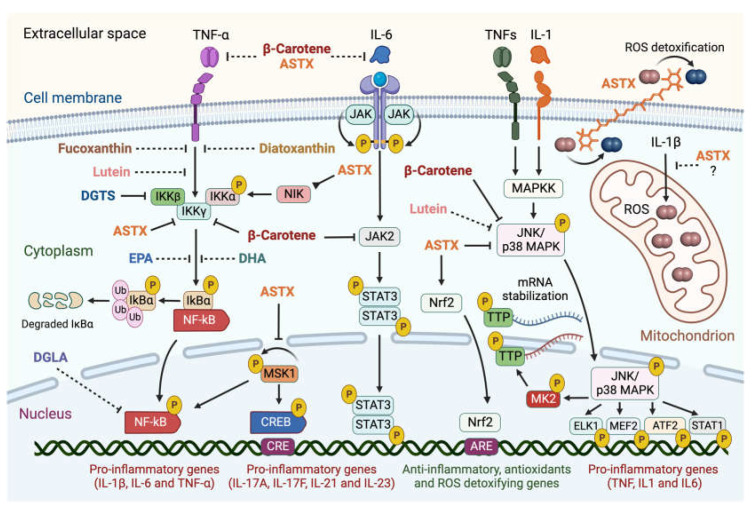
Summary of evidence-based interference of selected microalgal metabolites with the intracellular pro-inflammatory signaling pathways involved in the pathogenesis of RA. Microalgal carotenoids and lipids exert inhibitory effects on major intracellular pro-inflammatory signaling pathways involved in the onset and progression of rheumatoid arthritis. Blunt-ended solid lines indicate an inhibitory pharmacological effect, while dashed lines suggest proposed interference. Astaxanthin (ASTX), fucoxanthin, diatoxanthin, and β-carotene target different subunits of the Inhibitor of κB (IkB) kinase complex, preventing the phosphorylation-dependent release of the pro-inflammatory transcriptional activator NF-kB and its nuclear translocation. ASTX further acts upstream of this pathway through inhibiting the NF-κB-Inducing Kinase (NIK), preventing the autophosphorylation of the Mitogen- and Stress-activated protein Kinase-1 (MSK1), a nucleus-localized effector which activates NF-κB and the cAMP-responsive Element-Binding Protein (CREB) pro-inflammatory transcription factor. ASTX and β-carotene also inhibit the JNK/p38 MAPK signaling cascade, blocking the nuclear translocation of the JNK/p38 MAPK complex, and thus phosphorylation of downstream targets: the pro-inflammatory transcription factors ELK1, MEF2, ATF2, and STAT1, and the MAPK-activated kinase 2 (MK2) responsible for stabilizing IL mRNAs. β-carotene inhibits the JAK2/STAT3 pathway through blocking phosphorylation of the pro-inflammatory transcriptional activator STAT3 by JAK2 and its nuclear translocation. ASTX positively regulates the nuclear factor-erythroid 2-Related factor 2 (Nrf2)-mediated pathway involved in antioxidant and anti-inflammatory gene expression. ASTX and β-carotene are proposed to directly bind interleukin 6 (IL-6) and tumor necrosis factor-alpha (TNF-α), blocking their receptor interaction and activation of the downstream pathways. Lutein is suggested to inhibit both NF-kB and JNK/p38 MAPK pathways. ASTX detoxifies free radicals on both sides of the lipid bilayer and appears to suppress mitochondrial ROS production. The betaine lipid DGTS interferes with the activity of the IKKβ subunit of the IkB kinase complex, while the PUFAs docosahexaenoic (DHA), eicosapentaenoic (EPA), and dihomo-γ-linolenic (DGLA) acids modulate the NF-kB signaling cascade through targeting unknown pathway components. Figures created with BioRender.com, accessed on 15 November 2023.

Figure 4 summarizes the evidence-based interference of the above-mentioned microalgal carotenoids and lipids with the main intracellular pro-inflammatory pathways involved in the pathogenesis of RA. 

## 3. Bioprospecting and Domestication of Pharmacologically Relevant Microalgae

Of the over 70,000 estimated existing algal strains [164], only a few species are used in human nutrition and health. Bioprospecting for novel pharmacologically relevant species [165,166] requires scrupulous large-scale screening of phytoplankton biodiversity and chemodiversity [167], and the establishment of optimal cultivation strategies [168]. Inhospitable environments are excellent ecosystems to discover species with industrial applications since extremophiles are physiologically adapted to harsh conditions and hyperaccumulate pigments and lipids [11,12,169,170,171].

Among cryophilic species, the extracts of two Antarctic chlorophytes, *Chloromonas reticulata* (Chlorophyceae) and *Micractinium simplicissimus* (Trebouxiophyceae), were recently reported to exert an anti-inflammatory effect on activated macrophages [172,173]. At the other extreme, the rhodophyte *Cyanidioschyzon merolae* (Cyanidiophyceae) thrives at 40 °C and low pH, producing heat-stable carotenoids [174], while a stress-resilient strain of the marine chlorophyte species *Tetraselmis striata* [175] accumulates anti-inflammatory carotenoids and lipids [176]. A PUFAs-hyperproducing rhodophyte strain of the *Galdieria* genus (Cyanidiophyceae) was identified in acid thermal springs, and its lipid content could be enhanced via cultivation at temperatures below its optimal range [177]. Finally, the extracts of a chlorophyte *Mucidosphaerium* sp. (Trebouxiophyceae) isolated from a similar environment suppressed pro-inflammatory gene expression in human fibroblasts, as well as mitochondrial ROS production and inflammation in cultured synoviocytes [178,179].

### Turning Wild Species into “Unicellular Medicinal Crops”

Although the accumulation of therapeutic compounds in microalgae can be accrued through abiotic stress challenges [180,181,182,183,184,185], wild organisms are usually not suited for industrial applications. For instance, ASTX production via mass cultivation *H. pluvialis* is restrained by its slow growth and elevated risk of pest contamination [186,187].

**Figure 5:**
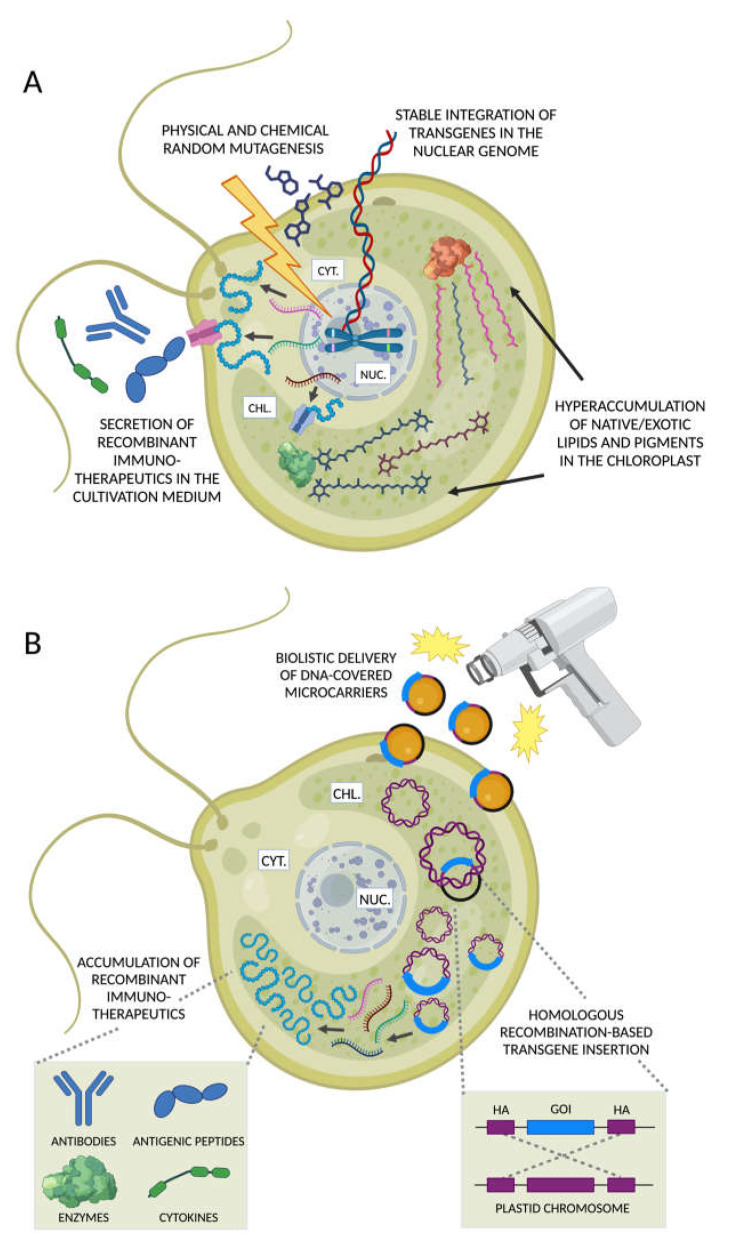
Nuclear and chloroplast engineering to produce immunotherapeutics in microalgae. (**A**) Foreign DNA sequences can be introduced in the nuclear genome of microalgae resulting in the stable integration of transgene(s). Trait evolution can be achieved through random mutagenesis using physical (e.g., UV radiation, violet flash) or chemical agents followed by the identification of phenotypes of interest. The recombinant protein products synthetized on cytoplasmic ribosomes can be either accumulated in the cell or secreted in the cultivation medium to facilitate their recovery. Alternatively, recombinant metabolic enzymes can be targeted to the chloroplast to enhance the biosynthesis of native long-chain polyunsaturated fatty acids (PUFAs), or even exotic metabolites with immunomodulatory/anti-inflammatory properties. (**B**) Transgenes are introduced in the chloroplast via biolistic delivery and targeted to defined chromosomal loci exploiting homologous recombination-enabled homology arm sequences (HA, purple) flanking the gene of interest (GOI, blue segment). The high copy number of plastid chromosomes ensures a significantly greater synthesis of different classes of recombinant immunotherapeutics compared with the engineering of the nuclear haploid genome. Figures created with BioRender.com, accessed on 15 November 2023.

Adaptive laboratory evolution and random mutagenesis are powerful strategies for strain improvement based, respectively, on spontaneous and enhanced mutation rates [188,189] (Figure 5A). This approach generated lipid- and carotenoid-hyperproducing strains of chlorophyte *Chlorella vulgaris* (Trebouxiophyceae) [190,191], enabling the discovery of new genetic targets to enhance lutein content [192]. Similarly, a simultaneous enhancement of ASTX and EPA was reported in the *Nannochloropsis* species *gaditiana* [193] and in the *Tetraselmis striata* [194], while DHA accrual was achieved in *Schizochytrium* sp. [195,196,197], *C. cohnii* [198], and *P. lutheri* [199]. It should be noted, however, that evolved strains are susceptible to the risk of retromutation and trait drift [200].

## 4. Biomanufacturing of Immunomodulatory Metabolites

### 4.1. Engineering Carotenoid Metabolism

Although microalgae can potentially substitute plants in the production of carotenoids like lutein [201,202], their yields are still lagging behind heterotrophic microorganisms [203,204,205,206]. Genetic engineering can greatly enhance pigment productivity in microalgae [207] but requires a detailed knowledge of algal genomes and of the transcriptional networks regulating carotenoid biosynthesis to manipulate key metabolic genes [208,209,210,211] (Figure 5A). In this respect, a model organism for ketocarotenoid biosynthesis is *C. zofingiensis* [212,213], from which the β-carotene ketolase (BKT) enzyme was identified as a rate-limiting factor for ASTX production [214], while the chlorophytes *D. salina* and *Desmodesmus* spp. (Chlorophyceae) are useful resources for β-carotene and lutein metabolism, respectively [215,216].

Carotenoid enhancement and the production of non-native ASTX isomers can be achieved through different engineering strategies [217], such as: (i) heterologous expression of chaperones stabilizing biosynthetic enzymes [218,219] and cyanobacterial proteins to improve pigment storage capacity [220], (ii) revival of endogenous silent *BKT* genes [221,222], and (iii) multigene overexpression to circumvent several biosynthetic bottlenecks [223]. Simultaneous enhancement of fucoxanthin and β-carotene was achieved with a similar approach in the diatom *Phaeodactylum tricornutum* (Bacillariophyceae) through overexpressing endogenous biosynthetic genes [224,225] and a plastoglobule protein to augment pigment sequestration [226]. Ketocarotenoids engineering was recently reported in the industrially relevant species *C. merolae* via heterologous expression of two biosynthetic genes [227], and in the thraustochytrid *A. limacinum* via overexpression of an endogenous β-carotene hydroxylase gene [228].

### 4.2. Synthetic Long-Chain Carotenoids

Algal chloroplasts provide excellent metabolic chassis to engineer the synthesis of non-native pigments with superior therapeutic properties [229], such as carotenoids with extended polyene chains and extra hydroxyl groups [230,231]. In this respect, extremophilic microorganisms are invaluable genetic resources to implement novel metabolic pathways in microalgae [232,233,234]. Archaea produce long-chain (C50) carotenoids through adding isoprene (C5) units to lycopene (C40) [235]. This biosynthetic pathway was successfully introduced in bacteria to produce C50 ASTX [236,237] and non-natural C60 ketocarotenoids [238], and it would be of extreme interest to verify its feasibility in microalgae to accumulate extremely valuable synthetic metabolites.

### 4.3. Enhancing PUFAs Accumulation

Microalgae can be engineered to maximize the yields of the endogenous anti-inflammatory lipids [239]. Overexpression of endogenous or heterologous fatty acid biosynthesis genes is a standard approach to enhance PUFAs productivity [240], and the availability of transcriptomes investigating stress adaptation is crucial to identify new factors to modulate lipid profiles [241]. For instance, *P. lutheri*, different *Prasinophyte* species of the genus *Ostreococcus* (Mamiellophyceae), and the diatom *Fragilariopsis cylindrus* (Bacillariophyceae) are model species for studying DHA and EPA biosynthesis [242,243,244]. Transcriptomics analysis of *Aurantiochytrium* revealed fatty acid synthase isoforms involved in DHA production under nitrogen starvation [245], while an acyl-CoA binding protein related to lipid droplet remodeling and EPA synthesis in *P. tricornutum* was recently suggested as a target to enhance the yields of therapeutic PUFAs [246].

Lipid enhancement was reported in the oleaginous chlorophyte *Neochloris oleoabundans* (Chlorophyceae) via heterologous expression of Kennedy pathway genes from the chlorophyte *Acutodesmus obliquus* (Chlorophyceae) [247], while DHA was increased in *P. tricornutum* through overexpressing the Δ*-6 desaturase* [248], and the heterologous Δ*-5 elongase* and *acyl-CoA-dependent* Δ*6-desaturase* from *Ostreococcus tauri* [249]. 

DHA enhancement was achieved in *Aurantiochytrium* sp. via overexpression of *glucose-6-phosphate dehydrogenase* to increase NADPH regeneration for fatty acid biosynthesis [250], and in a *Schizochytrium* sp. via overexpression of *ATP-citrate lyase* and *acetyl-CoA carboxylase* [251]. Lastly, EPA was increased in *D. salina* [252] with a heterologous *Δ-6 desaturase* gene from *T. pseudonana* [253]. 

Prostaglandin biosynthesis was engineered in the oleaginous diatom *Fistulifera solaris* (*Bacillariophyceae*), a natural producer of C20 PUFAs, via expression of a *cyclooxygenase* gene from the red macroalga *Agarophyton vermiculophyllum* (Gracilariaceae), resulting in the highest reported heterologous prostaglandin production in a photosynthetic host.

## 5. Production of Heterologous Immunotherapeutics in Microalgae

Microalgae can be genetically engineered [254] to produce heterologous biopharmaceuticals [255,256], including human immune-related proteins [257,258,259,260]. 

Nuclear transgenesis affords eukaryotic-like post-translational modifications of recombinant proteins (mainly N-terminal glycosylation) [261,262] and extracellular secretion to facilitate recovery [258,263] (Figure 5A). Although nuclear transgenesis results in low expression titers and requires intensive screening efforts to identify high-yielding strains [264], combinatorial transgene assembly and synthetic regulatory elements can greatly improve translation rates [265,266].

As in plants [267], the algal chloroplast genome affords high-level accumulation of recombinant therapeutics [268,269,270,271] (Figure 5B). The prokaryotic features of the polyploid chloroplast genome (plastome) enable efficient homologous recombination-based transgene insertion and multigene expression with synthetic operons [272]. A rich genetic toolkit allows plastome manipulation in non-model species [273], including marker-free and metabolism-dependent selection strategies [274,275,276]. Among these, the *type D phosphite dehydrogenase* (*PtxD*) gene, which enables the oxidization of non-assimilable phosphite ions in phosphate, is a biosafe solution to maintain axenic cultures [277,278], especially in mixotrophic conditions where pest contamination risk is high [279].

### Fast Tracking Microalgal Immunotherapeutics: The Time Is Now

Among newly proposed therapeutic agents for autoimmune diseases, fragment crystallisable (Fc) multimers against autoantibodies and peptides targeting Fc/Fcγ receptors are highly promising candidates [280]. Since microalgae can assemble full-length human monoclonal antibodies binding Fcγ receptors [281,282,283], it should be possible to produce antibodies targeting RA mediators [284]. Notably, polycistronic nuclear gene expression was reported in microalgae [285], potentially affording simultaneous production of multiple immunotherapeutic variants.

Viral nanoparticles are emerging tools with diagnostic and therapeutic applications in autoimmune diseases [286,287]. This strategy was pioneered in the plant *Nicotiana benthamiana* via self-assembled nanoparticles exposing RA autoantigens, which induced immunotolerance in animal RA models [288]. Although few studies have explored the use of viral vectors to produce recombinant therapeutics in microalgae so far [289,290], the ongoing characterization of microalgae-infecting viruses [291,292,293,294] should facilitate this approach in photosynthetic microbes [295,296].

## 6. Final Observations, Comments, and Outlook

Microalgae are key players in the transition towards a bioeconomy [297], and are the focus of intense academic and private research [298,299]. In Europe, the number of companies engaging in microalgae production is growing [300], and so is the list of patents describing their therapeutic uses in inflammatory diseases [301]. Microalgal biotechnology, however, is still restrained by low product yields and recovery [302,303,304,305,306], high operating costs, and barriers to market entry [307,308], especially for engineered strains [309].

### 6.1. Building a Good Reputation

Arguably, the biggest obstacle to the nutritional uses of photosynthetic microbes is their recognition as safe [310]. Currently, only five eukaryotic microalgae and three cyanoprokaryotes hold Novel Food status [311]. The occasional finding of hazardous contaminants such as heavy metals, toxins, pathogens, and pesticides in the harvested algal biomass [312,313] calls for rigorous adherence to good manufacturing practice in the microalgal food industry [314,315], especially in the case of emerging strains [316]. An even greater barrier is the fear of horizontal transfer of antibiotic resistance genes from engineered strains to the gut microbiome and human pathogens [317], thus the adoption of marker-free selection [274] and metabolic markers [275] are expected to promote a more favourable attitude towards engineered microalgae and their derived products.

### 6.2. A Road Map for Clinical Uses of Microalgae in Chronic Inflammation

To advance the applications of microalgae in autoimmune diseases, the therapeutic potential of novel classes of bioactive compounds should be explored. For example, some microalgal strains are sustainable sources of the lipid-based prohormone vitamin D_3_, the biosynthetic precursor of the biologically active 1,25-dihydroxyvitamin D (calcitriol) [318,319] whose deficiency is a major risk factor for RA [320,321]. Several microalgae accumulate bioactive ergosterol and β-sitosterol [322,323], precursors of vitamin D_2_ (ergocalciferol) and D_3_, respectively [324,325]. Moreover, the chlorophyte *Botryococcus braunii* (Trebouxiophyceae) and *Schizochytrium mangrovei* can be engineered to hyperaccumulate squalene, an anti-inflammatory triterpene and precursor of ergosterol [326,327,328,329,330,331].

The photosynthetic protist *Euglena gracilis* (Euglenoidea), a recently authorised Novel Food species [332], also deserves attention, being a natural producer of anti-inflammatory carotenoids, DHA [333,334,335], and paramylon, an immunomodulatory (1,3)-β-glucan [336] with reported inhibitory activity towards Th17 cells [337].

Finally, photosynthetic prokaryotes are still largely unexplored biomanufacturing platforms, despite producing a vast repertoire of unique compounds [338], including molecules with antioxidant and anti-inflammatory properties [339,340] like the pigment–protein complex phycocyanin, which is a selective inhibitor of pro-inflammatory oxylipin synthesis [341,342], and various polysaccharides [343,344,345] and peptides [346,347,348]. Last but not least, an increasing number of genetic engineering and synthetic biology tools [349] can be employed to produce recombinant therapeutics in cyanoprokaryotes [350], as already reported for immunomodulatory proteins [351].

## 7. Conclusions

The exploration of microalgal biodiversity and chemodiversity is a promising approach to discover new complementary therapeutic approaches for the management of RA and related chronic inflammatory conditions. Indeed, several preclinical studies have highlighted multiple mechanisms of action of microalgal compounds and their interference with pro-inflammatory pathways. Moreover, the availability of advanced genetic engineering tools holds tremendous potential to develop innovative biopharmaceuticals in photosynthetic microbes and expand their clinical applications.

## Data Availability

No new data were created or analyzed in this study.
